# Image-Guided Navigation in Spine Surgery: From Historical Developments to Future Perspectives

**DOI:** 10.3390/jcm13072036

**Published:** 2024-04-01

**Authors:** John Preston Wilson, Lane Fontenot, Caleb Stewart, Deepak Kumbhare, Bharat Guthikonda, Stanley Hoang

**Affiliations:** Department of Neurosurgery, Louisiana State University Health Sciences Center Shreveport, Shreveport, LA 71103, USA; jpw002@lsuhs.edu (J.P.W.J.); lane.fontenot@lsuhs.edu (L.F.); caleb.stewart@lsuhs.edu (C.S.); deepak.kumbhare@lsuhs.edu (D.K.); bharat.guthikonda@lsuhs.edu (B.G.)

**Keywords:** intraoperative navigation, image-guided navigation, CT image-guided navigation, fluoroscopic navigation, minimally invasive spine surgery navigation, robot-assisted image-guided navigation, machine vision image-guidance, spine surgery navigation, pedicle screw fixation

## Abstract

Intraoperative navigation is critical during spine surgery to ensure accurate instrumentation placement. From the early era of fluoroscopy to the current advancement in robotics, spinal navigation has continued to evolve. By understanding the variations in system protocols and their respective usage in the operating room, the surgeon can use and maximize the potential of various image guidance options more effectively. At the same time, maintaining navigation accuracy throughout the procedure is of the utmost importance, which can be confirmed intraoperatively by using an internal fiducial marker, as demonstrated herein. This technology can reduce the need for revision surgeries, minimize postoperative complications, and enhance the overall efficiency of operating rooms.

## 1. Introduction

In spine surgery, methods for increasing precision and safety are sought after by both surgeons and industry. Image-guided spine surgery has become a crucial breakthrough for advancing patient safety and surgical efficiency. Since the mid-1990s, the maturation of image-guided spine surgery has provided a platform for expansion in the techniques of spinal oncology, minimally invasive surgery, deformity correction, and other approaches [1]. Image-guided navigation technology in spine surgery is becoming increasingly common in many medical facilities [2,3]. A recent study investigated 183,620 neurosurgical patients undergoing spinal fusions from 2003 to 2019. The study revealed a notable increase in image-guided assistance, with 10.76% of all fusions implementing navigation in 2017, compared to only 0.38% in 2010 [4]. Another study investigating the utilization of computer-assisted navigation found that 16.8% of fusions were image-guided, with higher implementation rates in academic settings [5]. The rapid advancements in image-guided technologies and the increase in access to this technology will broaden the use of image-guided techniques over the years. Further advances require close collaboration between the surgeons and the technical team, as the surgeons must use their expertise to determine the practicality of the technology. They also play a crucial role in regulating and creating new systems to ensure advanced technology is helpful in the operative setting.

With the rapid advancement of medical technology, it has become critical for surgeons to broaden their training beyond traditional techniques. Navigation-assisted surgery is an example that requires surgeons to not only possess exceptional surgical skills but also extensive knowledge of instrumentation and computer guidance systems. As surgeons become more familiar with image-guided navigation systems, they are more attuned to their capabilities and can provide input into how the technologies can be adapted. Innovations in medical technology continue to emerge throughout the entirety of health care, with image-guided navigation and surgical preplanning standing at the forefront of what has been defined as “digital surgery” [6]. 

The spine’s complex anatomy and delicate nature require advanced imaging modalities. Before the inception of image-guided navigation, patient-specific anatomy prompted intraoperative visualization by exposure to perform procedures. However, the rapid development of computer systems technology has allowed new and real-time partnerships to flourish in the operating room. Image-guided navigation has significantly increased its applications in spine surgery since its onset. Image-guided navigation technology is critical for improving surgeons’ visual capabilities as they operate. Improvements to the surgeon’s visual field can enhance a range of measured surgical metrics related to an increase in accuracy for instrumentation and an increase in the efficiency of the operative workflow. From an administrative and patient safety perspective, any technology that reduces postoperative complications and improves operating room workflow is valuable to both parties. As such, technological advancements in neurosurgery have resulted in cooperation between surgeons and technical fields. The interactions between the two can include situations where the surgeon drives innovation in the technical world. Conversely, advances in the technical world can be the driving force behind updated clinical practice. As technological advancements occur, the scale shifts towards technology, shaping surgical techniques.

Understanding the development of image-guided navigation is helpful for surgeons in translating the latest technology to current treatments. This understanding allows surgeons to optimize the existing capabilities, anticipate limitations, and determine protocols that dovetail treatment with biomedical devices. Surgeons exemplifying this level of technological fluency will have an advantage by allowing full engagement with the technology, enabling them to contribute to further advancement while setting the curve for procedural trends. The surgeon’s role is evolving from merely passively receiving technological advances to a need for active participation in the innovation ecosystem. Surgeons, as end-users, provide invaluable insights that can guide the development of more advanced, intuitive, and efficient navigation.

## 2. Historical Overview of Image-Guided Spine Surgery

Before the advent of imaging technology, the early years of spine surgery solely relied on stereotaxis, which included surface-mounted reference points and fixed external frames. Early attempts at using stereotaxic principles in the upper cervical spine were introduced in 1967 using a modified Leksell frame [7]. As expected, the clinical outcomes were unpredictable and highly unfavorable due to a lack of robust visual references. Even with the introduction of computed tomography (CT), surgeons were limited to the postoperative window to determine instrumentation accuracy. A solution to this hurdle was introduced with the concept of intraoperative imaging. The path to image-guided navigation and where it exists today was underway, and radiographic images captured intraoperatively were the foundation of this new concept.

The need to visualize anatomical landmarks led surgeons to seek various methods, including lateral plain radiography, in the 1970s, 1980s, and 1990s. Lateral radiographs were used primarily in pedicle screw placement and internal fixations [8,9,10]. Other techniques around the time employed CT imaging with stereotactic systems and fluoroscopy to localize anatomic structures and identify lesions [11]. However, a shift in the approach to intraoperative visualization was introduced when the first case using “frameless stereotaxis” was employed in 1994. This group was the first to apply modern image-guided technology to spine surgery by utilizing three-dimensional (3D) multiplanar imaging of the lumbosacral spine to determine screw entry points [12]. 

### 2.1. Evolution of Imaging Modalities

The first imaging modality, X-ray, was introduced by Wilhelm Roentgen in 1895 [13]. Its discovery was accidental, resulting from an unrelated experiment. Roentgen observed lasting images from cathode rays passing through an induction tube. His investigation centered around the mathematical properties of X-rays passing through substances of different densities or thicknesses of material. This led to the development of the “X-ray,” which paved the way for image guidance in spine procedures. Numerous stages of development would need to be explored to make the valuable technology applicable in the operating room. Using lateral radiography to confirm screw placement or guiding surgeon trajectory in the 1970s marked the onset of using imaging modalities for navigation assistance. The next step in the development path to 3D navigation was the creation of image intensifiers and burst radiography. These techniques expanded the understanding of radiation intensity and revolutionized surgical procedures by increasing the light intensity of the fluoroscopic image. In the 1990s, optimizing this technique and associated image distortions in the operating room involved exploring the methods behind targeting necessary anatomy for treatment planning [14]. On the technical side, optimizing image-intensifier systems included deriving the optimal radius of curvature of the input screen, view angle, distance from the focal spot, and other factors [15]. However, even with improved imaging modalities, the surgeon was still required to fully expose the operative field and identify the targeted region in the vertebral column. 

The success of first-generation navigation systems in the early 1990s demonstrated the potential for reducing radiation exposure and other drawbacks associated with intraoperative fluoroscopy [16]. To overcome some of these hurdles, CT imaging, first used in 1971 on a live patient, offered a more reliable visualization [17]. Compared to traditional fluoroscopy methods, CT navigation minimized occupational radiation exposure, enabled surgical visualization without direct sight, and provided more ergonomic setups [18,19]. The evolution of imaging modalities provided essential advantages in accuracy, visualization, and surgical workflow, making navigated spine surgery viable for widespread adoption [18,19,20]. Transitioning from preoperative to intraoperative CT conferred additional benefits, such as automated registration methods and confirmatory imaging for hardware position [21]. Magnetic resonance imaging (MRI) arose during the 1970s and produced images with advanced discrimination of soft tissues and anatomical relationships. MRI has the advantage of not requiring ionizing radiation to produce high-resolution images. Although MRI has allowed for improved visualization of intervertebral discs and endplates, there are limitations associated with the technology that prevent more regular use intraoperatively [22].

### 2.2. The Impact on Surgical Techniques with Evolving Navigation Technology

With the advancement of image-guidance technology, a surgical specialty that once relied on preoperative imaging, fluoroscopy, and stereotaxy was entering an era of presurgical planning and improved precision rates. While previously established imaging modalities such as fluoroscopy still have roles in today’s surgical practice, introducing 3D imaging has helped increase instrumentation accuracy and decrease operative time. Without developments made to preexisting technology, the inception of 3D imaging would be unlikely. The basis for which these systems operate is by using registration techniques with patient imaging, either CT, preoperative or intraoperative, or intraoperative fluoroscopic images, and uploading them to imaging software in the operating room. Two-dimensional (2D) fluoroscopic navigation principles laid the groundwork for building the more visually encompassing model of patient anatomy with isocentric 3D fluoroscopic navigation. Both methods incorporate foundational fluoroscopic techniques for patient navigation practices [23]. 

**Figure 1:**
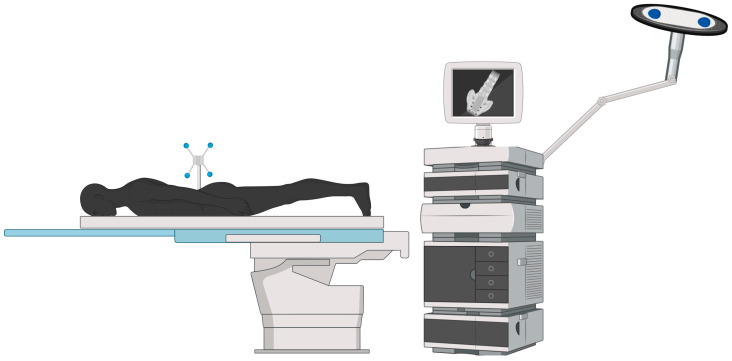
Dynamic reference array configuration: This image demonstrates the utilization of a dynamic reference array (DRA) to exhibit a patient’s anatomy. The DRA is affixed to the patient and identified by the camera through the attached blue reflective markers. Additionally, the workstation and camera are depicted alongside the operating table to show the workstation setup relative to the patient.

Image-guided navigation initially relied on an image registration strategy called “paired-point registration” [24,25]. This approach to navigation involves selecting points on the patient’s 3D-constructed anatomy generated from preoperative imaging and then touching that point to the patient’s actual anatomy [25]. The limitation of this approach is that there is room for surgeon error in the registration stage of the protocol; additionally, pairing with the patient’s anatomy requires extensive exposure and prevents this technique from being implemented in minimally invasive procedures. Limits and application boundaries aside, this technique has reported success in pedicle screw fixations [8,26]. Another approach to the stereotactic image guidance is with a registration device known as a dynamic reference array (DRA). DRAs are fixed to the patient, and reflective spherical markers allow navigation system cameras to track the location and, later in development, the movement of the patient’s anatomy [27]. A graphic example of the tracking system synced to a reference frame can be found in Figure 1.

When discussing the clinical advantages of navigation in spine surgery compared to traditional methods, the improved rates of clinical outcomes must be considered. Compared to conventional surgical techniques, each new age in navigation technology has generally resulted in more accurately placed instrumentation. A comprehensive meta-analysis investigated traditional surgical practices’ clinical outcomes compared to computer-assisted navigation outcomes. From 1966 until 2006, 37,337 total implanted pedicle screws with navigation assistance and without navigation assistance were included in the study [28]. The study found that 34,107 pedicle screws (91.3%) were placed accurately. However, the two subgroups consisting of navigation-assisted and conventional methods revealed the difference in accuracy between the two processes. In the computer-assisted subgroup using isocentric 3D fluoroscopic navigation, the median reported accuracy was 95.2%. Comparatively, conventional methods without navigation demonstrated a median accuracy of 90.3%. 

The preliminary navigation assistance systems discussed previously formed the foundation for the next-generation navigation-guided procedures. Although more favorable outcomes were achieved with the primary methods and subsequent developments shortly after that, the limitations of the techniques and prolonged operating times with cumbersome image registration slowed the widespread adoption of navigation assistance. In the systems subsequently developed, there was a more favorable outlook on the future of image-guided surgical navigation. In 2006, the cone beam CT was developed with the embodiment of the O-arm navigation system, which reconstructed multiplanar images. This was the first step towards the advanced technologies centered around robotic and machine vision navigation [29,30].

## 3. Current State of Image-Guided Spine Surgery

The necessity to offer the highest standard of patient safety, coupled with the continued advancement in computer systems and medical technology, has resulted in exponential growth and development of image-guided navigation. Various medical technology companies have entered the business of developing these systems with input from surgeons, hoping to continue developing the technology even further. As different systems implement different strategies in their race to create the most robust image-guided navigation system, there exists a variety of choices. However, due to the range of advancements in the various modern systems, the necessary infrastructure to employ these platforms, and the education needed to understand best-case uses fully, it is essential to plan and comprehend image-guided functionality within the different platforms to maximize patient outcomes. 

### 3.1. Methods for Image-Guided Navigation

Most modern navigation systems have standard components. They include a computer workstation with system software that displays 3D anatomy [31]. Additionally, in systems with optical tracking, an infrared camera is attached to the system to register and map surgical tools. These components form the foundation of many modern navigation systems. As previously discussed, the differences in system components for navigation systems come from the various options available and the companies that provide them. Today’s growing range of general image guidance and navigation systems include previously developed concepts and state-of-the-art techniques. Some examples of older imaging technologies include fluoroscopy-based and CT-based navigation, which can be either 2D or 3D [32,33,34]. Although these technologies were developed before more advanced techniques implementing machine learning and robotics, their usefulness in the operating room has retained clinical value due to continued software and biomedical engineering development. Next-generation image-guidance technologies are, however, beginning to implement novel components that are integral to the functionality of these systems. Examples of novel components made available with recent advancements can be found when discussing platforms built on optical navigation, robotic-assisted image-guided navigation, advanced image processing, machine learning navigation, and augmented reality systems [35,36,37,38,39]. 

### 3.2. Active versus Passive Navigation in Image-Guided Surgery

The technology of image-guided navigation can be classified as active or passive, depending on the surgeon’s involvement. Active systems have elements that either direct the surgeon or use external fixations, like robotic arms, to guide the surgeon’s movements. Systems considered active navigation are restrictive to surgical movements that deviate from predetermined paths set in the presurgical planning stage of the procedure. Image-based systems incorporating active navigation components, such as in robotics or drilling guides, utilize a predetermined trajectory set during the preoperative planning stage of the process [40]. 

Unlike active navigation systems, passive technology allows the surgeon more flexibility and does not restrict movements. Instead, these tools visually represent the surgical trajectory path. Passive navigation systems aid the surgeon in keeping their attention on the intended surgical location while allowing for the adaptability to modify their trajectory depending on obstacles that present with the patient’s physical makeup. Passive 3D navigation platforms, such as CT navigation, offer a wide range of procedures to which they can be applied. These include deformity correction, trauma surgery, spinal tumors, spinal stabilization, and minimally invasive approaches [41]. All these procedures require precise and versatile movements. 

Irrespective of the image-guidance navigation system employed in any given procedure, the reduction in human error by careful surgical planning and proper implementation of protocols with these systems is of utmost importance. One study investigating pedicle screw accuracy in treating idiopathic and degenerative deformities revealed that the accuracy progressively deteriorates with increasing distance from the DRA [42]. This finding highlights the importance of proper preoperative planning and careful consideration of the placement location of the reference frame.

### 3.3. Two-Dimensional versus Three-Dimensional Navigation

It can be challenging to distinguish between modern image-guided modalities and their navigation techniques, as they share similar components. However, with proper training, it can be easier to determine the best way to use them. Differences in navigation technologies can be identified when examining the modality used for guidance, the imaging protocol, and the timing and manner of registration. Additionally, with more advanced systems that integrate virtual surgical tools for tracking, education on the system before use is needed. 

Using fluoroscopic or CT-based navigation has a long past and remains a common practice. However, with the emergence of more innovative and sophisticated systems, it is crucial to grasp present-day technology to recognize the transformative impact of traditional navigation assistance on the industry. The application of fluoroscopy for spine surgery navigation offers a 2D view of the patient’s anatomy. This is accomplished with a traditional C-arm capturing lateral and AP images of the patient. This typically involves introducing and removing the C-arm throughout the procedure. Pitfalls of this navigation modality relate to the timing of which imaging is collected. Fluoroscopic imaging provides more of a general guide to the anatomic structures targeted or confirmation of instrumentation placement.

Moreover, current clinical practice has identified CT-guided navigation as superior to fluoroscopic navigation [34]. The reoperation rates between the two modalities are not significant. However, the accuracy of pedicle screw placement is markedly higher with CT navigation. The approach to CT image-guided navigation generates 3D multiplanar imaging with intraoperative CT scans [32,34]. Due to the inherent differences in radiation exposure experienced between fluoroscopic and CT imaging, there has been significantly higher radiation exposure in CT navigation-guided procedures [43]. However, most CT scanners can use a low-dose radiation exposure protocol for imaging. This can aid in reducing the patient’s radiation exposure [44,45]. The associated increase in radiation exposure arises from the inherent nature of CT navigation protocol, which typically consists of a confirmatory spin for checking instrumentation. Although the risk associated with increased radiation exposure is well understood, surgeons must balance this risk with the added benefits of CT navigation. A research study comparing fluoroscopic navigation and CT navigation for treating degenerative lumbar stenosis revealed that CT navigation boasted a higher accuracy rate of 95.5% compared to the 91.5% accuracy rate of fluoroscopy techniques [34]. The visual 3D reconstruction of patients’ anatomy with CT navigation allows for increased rates of accuracy for pedicle screw insertions. Additionally, CT navigation has not been found to have negatively impacted operating room workflow, maintaining the same level of efficiency as with fluoroscopic navigation. Success for the fixation of thoracolumbar fractures using CT navigation has also been described [46]. CT navigation techniques have demonstrated decreased rates of pedicle cortical breach compared to traditional freehand surgical methods in different studies [47]. One study investigating institutional outcomes following 329 procedures found that intraoperative C-arm fluoroscopy is crucial to improving patient outcomes [48].

### 3.4. Advanced Systems and Clinical Results

Modern 3D image-guided navigation systems are rapidly improving by integrating advanced methods built into robotics, optics, machine vision, and augmented reality platforms. These systems incorporate advanced programs that contain shared components from the conventional 2D and 3D imaging modalities. The added benefit of these methods is that they offload risks associated with human error. This can be achieved by increasing the workload on machine systems to maintain trajectory, enhancing computer vision and surgical tool tracking, and using robotic precision and augmented reality for digitalization. 

**Figure 2:**
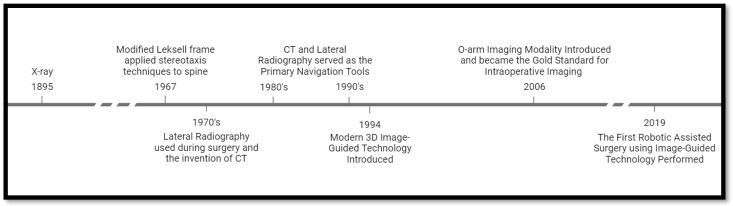
Navigation development timeline: This figure displays the development of pertinent imaging technology that paved the way for the modern intraoperative navigation guidance systems surgeons use today.

The field of spinal navigation has a strong competitor in robotic-assisted systems. Early robotic-assisted methods filled the surgical assistant role by providing the surgeon with increased accuracy and predefined trajectory paths by incorporating preoperative planning software. Robotic assistance coupled with navigation systems is rapidly developing and becoming more widespread in operating rooms. This is due to increased awareness and training on how to use them effectively. The first spine surgery to implement real-time image-guided robotic assistance was performed in 2019 [49]. Reduced surgeon fatigue, improved pedicle screw accuracy, decreased radiation exposure, increased potential applications in minimally invasive surgery, and reduced training time have been reported. The advancements in navigation-guided technology can be traced back to earlier techniques and achievements. Figure 2 displays a timeline showcasing these advancements that have led to the current state of the technology. This milestone in navigation-guided technology represents a significant progression from earlier techniques, and it exemplifies the dedication and hard work of many experts in the field.

The first image-guided robotic-assisted surgery secured the robot to the floor with an outstretched arm for transpedicular drilling and screw placement. This was not only the first instance in which this advanced next-generation navigation system was used, but it also resulted in a favorable postoperative outcome. Not long before establishing success with a combined robotic image-guided navigation system, there was only stand-alone robotic assistance. However, these methods introduced increased radiation exposure, trajectory failure, and other pitfalls that were primarily addressed with the coming together of robotics with navigation. Even with the limitations associated with stand-alone robotic assistance, studies have reported favorable results regarding the accuracy of pedicle screw insertions in the range of 94.5% and 99% [37,40,50,51,52]. One study investigating floor-mounted robotic pedicle screw placement found pedicle screw accuracy in 229 patients with 1050 screws to be 96.4% [53]. As software programs and available technology continue to advance, surgeons will adapt. 

Another technology that was early in its utilization was a machine vision image-guided system. Optical topographic imaging (OTI) is considered one of the up-and-coming image-guided navigation technologies being developed further and slowly adopted by surgeons in today’s operating rooms [35]. This system operates by projecting light patterns onto the patient’s skin, which will be further overlayed with preoperative CT imaging. This modality requires that there be sufficient detection of patterning for further recognition by the OTI system. This method allows surgeons to obtain highly accurate 3D scans of a patient’s bony anatomy without exposure to harmful radiation. This cutting-edge technology allows these scans to be automatically registered to preoperative CT scans, enabling surgeons to navigate with precision and accuracy without further intra-operative scanning. This eliminates the need for additional radiation exposure and further streamlines the surgical process, allowing for faster and more efficient procedures [54]. This technology was successfully implemented for the first time in 2022 for the surgical treatment of a traumatic unstable thoracolumbar fracture. The use of machine vision navigation has the potential to reduce radiation exposure for both patients and surgical staff significantly. 

Augmented reality technology is another novel component used in new systems that incorporates surface scanning technology to offer a slightly alternative approach for bypassing surgeon error. With an augmented reality system, optical fiducials are attached to the patient’s back and include holographic images for the surgeon to manipulate to align the position of screws. This image-guided system requires the surgeon to wear a virtual reality headset to view the holographic images. Optical self-adhesive tags bring the screen with surgical planning over the patient. Using image registration and virtual pathways, the surgeon can align the trajectories for the screws down the middle of the pedicle in both the sagittal and coronal planes. Augmented reality image guidance was first used successfully in 2022 [55]. The result of attempting this new navigation technology on cadaveric specimens demonstrated an accuracy of 96%, higher than previously discussed conventional freehand or fluoroscopic techniques. This approach to navigation has benefits tied to improved visualization and decreased operating time and blood loss. Surgical resection of intradural extramedullary tumors has also successfully been completed using AR [56]. AR has also been used for transforaminal lumbar interbody fusion, with one study supporting its use following ten successful technology implementations [57].

Spine surgery can lead the way in advanced medical technology, as shown by the rapid development of image-guided navigation systems. While there are still technical challenges to overcome with existing systems like robotic-assisted image guidance and 3D CT navigation and emerging technologies such as machine vision and augmented reality, the future looks bright. Spine surgery benefits from more efficient workflow, improved patient safety, and greater accuracy in instrumentation. 

## 4. Technical Overview of Image-Guided Navigation

Understanding the progressive development of these systems is instrumental in allowing surgeons to continue to serve an essential role in the continued advancement of navigation systems. Equally as important are the technical inner workings of commonly employed systems. The opportunity for continued advancement in image guidance navigation is higher when surgeons maintain a level of understanding beyond the role of the end user. As new-age systems incorporate robotic components, augmented reality, and virtual reality, the bulk of spine navigation uses CT-based image guidance systems such as the Medtronic StealthStation S8 Surgical Navigation System (Medtronic, Minneapolis, MN, USA). Regardless of the company providing the system, the inner workings of image-guided navigation systems can be viewed through four objectives: preoperative planning and 3D reconstruction, image registration and accuracy verification, dynamic instrument tracking, and visualization.

For preoperative planning in CT-based systems, the patient will receive a high-resolution CT scan in the operating room using an O-arm or similar device to verify the patient’s anatomy and upload to the computer workstation. The 2D image slices obtained by the CT machine are converted to a 3D model using a volume rendering algorithm [58]. Volume rendering, or creating a 3D anatomic model, also known as a digitally reconstructed radiograph (DRR), involves using advanced algorithms such as ray casting. These algorithms send rays through the 3D data volume and map the various pixels based on differences in color, opacity, and tissue density [59]. To help algorithms correctly map the 3D reconstructions, threshold points for the different colors and opacities are employed to isolate the various anatomical structures. The result of these algorithms provides a 3D model for screw planning and for navigation guidance. The 3D models can then be used to aid spine surgeons.

After 3D reconstruction, CT-based image guidance systems must complete registration with patient anatomy and verification of accuracy. This is accomplished using reference frames and DRAs to align anatomical landmarks in the physical space with their corresponding point on the 3D model. Navigation systems complete this step by implementing point-based and surface-based registration methods. In point-based registration, an iterative closest point (ICP) algorithm operates to minimize the distance between point sets. This is conducted by manipulating the rotation and translation of corresponding points between the patient and the 3D image. By controlling the location of these points in space, ICP algorithms work to fuse the scan and 3D intraoperative image, completing the registration and verification stage [60]. Surface-based registration methods operate similarly to minimize distances. However, these algorithms gather a group of points from the surgeon on the patient’s surface and adjust the model to align with these points.

One of the more impressive components of image-guided navigation systems is the ability to have dynamic navigated instrument tracking. Either optical or electromagnetic tracking provides the ability to track surgical instruments. Electromagnetic tracking, using sensor localization algorithms, calculates the sensor’s position and orientation, which model the interaction of the disturbances between the sensors and instruments’ field [61]. The optical tracking method is commonly used, employing strategies of feature detection and stereovision and matching algorithms. Feature matching operates to detect features of registered instruments and matches frame-by-frame movement. The other optical tracking technique involves cameras that view the surgical field and instruments. Identifying the reflective markers on reference arrays is followed by triangulating their location in the operative space. Stereovision algorithms track the points of reference arrays by mapping the coordinates of intersecting lines between the two cameras. These techniques simulate the movement of surgical instruments within the 3D environments in real-time, allowing surgeons to see the relationship between surgical tools and the patient.

**Figure 3:**
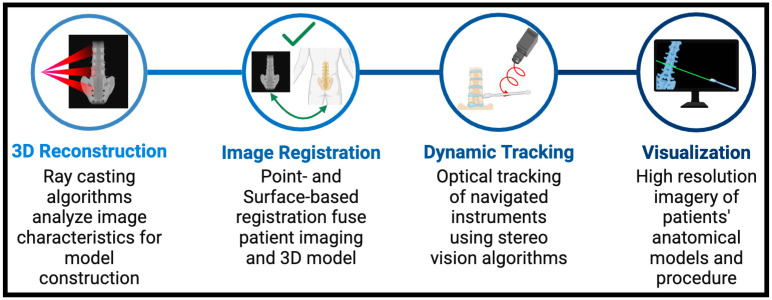
Technical operations of CT-based navigation: This figure shows the functional components that allow image-guided navigation systems to operate. Beginning with 3D reconstruction, shown in the first panel, CT-based systems develop an anatomical model using preoperative patient imaging. The 3D model is then used to register the patient’s anatomy in space, synchronizing the model’s orientation and size with the patient. Intraoperative tracking of surgical instruments is achieved using cameras that provide information on the location of reflective markers in the operative space. Finally, high-resolution visualization of the patient’s anatomy and surgical instruments throughout the procedure is accomplished with various application program interfaces that process changes as they develop.

Lastly, to complete the functionality of CT-based image guidance systems, there is a need for high-resolution real-time visualization of the 3D model and associated instruments. Navigation systems will utilize affine transformation matrix computations that transform the coordinates of tracked instruments and changes in patient positioning into the corresponding coordinates within the 3D model space. For real-time rendering of these dynamic changes, an open graphics library (OpenGL) or DirectX application program interface works to actively update the system projections on the viewing screen. These functions work together to make 3D models and their interactions with surgical instruments accurate and smooth throughout the procedures. The overarching objectives shared between CT-based image-guided navigation systems and the respective means to accomplish them are shown in Figure 3.

## 5. Clinical Workflow Using Navigation-Guided Technology

**Figure 4:**
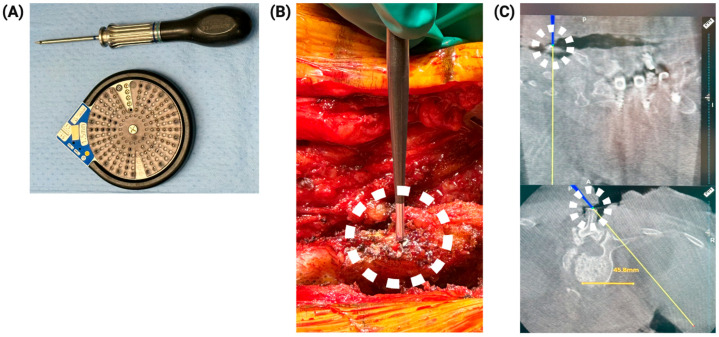
Accuracy confirmation using navigation: The Medtronic Stealth Navigation System accuracy is confirmed with internal fiducials. (**A**) Shows the Stryker screws used. (**B**) Displays the navigated instrument making contact with the Stryker screw on the patient’s spinous process in the surgical field. (**C**) Displays the navigated instrument making contact with the Stryker screw (metallic hyper-density) on the intraoperatively obtained CT images. The accuracy demonstrated here is excellent.

The foremost concern with navigation technology is ascertaining accuracy throughout the operation. Here, we discuss the workflow at our institution and a method to help confirm accuracy by placing an internal fiducial. For posterior cervical cases, the patient is generally fixed in a radiolucent three-point Mayfield. The patient reference frame can be placed on the spinous process or attached to the Mayfield. The latter is especially suited for occiput and high cervical procedures. For lumbar and thoracic cases, the reference frame is placed on the spinous process or in the posterior superior iliac spine (PSIS). In the case of a PSIS pin for a lumbar procedure, following appropriate draping, the PSIS is identified using a spinal needle. The pin is then placed, ensuring the proper angle for enough working room. Consideration of an adequate view for the navigation cameras to detect the reference frame is critical here. To place the internal fiducial, after adequate exposure, a 4mm Stryker screw (Stryker, Kalamazoo, MI, USA) is placed in the superior level and lowermost spinous processes for accuracy feedback. Placing the fiducial before intraoperative scanning with the O-arm allows for manual matching and confirmation of anatomic accuracy. We have found the metallic fiducial more visually reliable in confirming accuracy than the native bony landmarks. An example of this method is shown in Figure 4. The intraoperative CT is obtained with the O arm for navigation reference imaging. To help maintain a sterile field, two sterile quarter sheets are placed widely over the sterile area and stapled together with an exit hole for the navigation frame. The frame is typically covered with a sterile towel when the O arm is brought into the field. Once the O-arm is in position, the sterile towel is removed. This process aids in maintaining a sterile working field.

## 6. Conclusions

Image-guided navigation has been revolutionary to how spine surgeons approach their procedures. Since image-guided systems allow for visualization of patients’ anatomy, they are, for all intents and purposes, essential for the surgeon’s ability to minimize human error. Malpositioned pedicle screws can lead to highly unfavorable outcomes, leaving the patient with neurological deficits such as radiculopathy, myelopathy, dural tear, epidural hematoma, and pedicle screw fracture due to poor instrumentation [62]. A 2018 study discovered that up to 10% of pedicle screws placed using freehand techniques are malpositioned [63]. Additionally, they revealed that 1 in 300 patients may undergo revision for these malpositioned pedicle screws. The incidence of malpositioned screws could decrease if image-guided navigation were implemented in all spine procedures. Surgeons may only use navigation assistance in certain instances because of the increased cost of using these systems. A 2015 study of a sample of cases in which navigation was used found a range of costs associated with the technology, anywhere from USD 17,650 to USD 39,643 [64]. Nonetheless, the cost-effectiveness of using intraoperative navigation has been reported to be highly beneficial for the surgeon and hospital by reducing reoperation rates and postoperative complications [65]. One limitation of the technology is the time, cost, and educational resources needed to personalize learning environments and integrate upcoming physicians with developing technology [66,67]. The difficulties regarding adequate education and training that neurosurgery residents face globally are exacerbated by advanced technologies such as those discussed in this manuscript. This is partly due to the limitations of the available means to access related technologies or the lack of necessary infrastructure, which can include knowledgeable technicians who service these platforms.

As the advancement of technology drives on, the eventual reduction in costs will arise as systems become more capable, widely accepted, and efficient. Specific examples, such as machine vision, robotic assistance, and augmented reality, will decrease their utilization cost as competitors in the market develop more cost-effective solutions. Advanced techniques such as these will further improve navigation by minimizing the surgical interference and interventions that may arise or are needed to register the different systems properly. Surgeons can anticipate numerous changes in the field of spine surgery due to advancements in technology, particularly in navigation. Since the introduction of X-rays, spine procedures have evolved to include more advanced systems such as 2D-fluoroscopy or 3D-CT navigation, robotic assistance, machine vision, and augmented reality. The evidence shows that navigation assistance is crucial in achieving unmatched precision and providing excellent patient care. These developments highly influence the ever-evolving landscape of this field. Future directives for making navigation technology a standard of care should focus on reducing its cost, enhancing education and training resources, and continually advancing the existing systems.
